# A rare case of a swollen knee due to disseminated synovial chondromatosis: a case report

**DOI:** 10.1186/1752-1947-4-113

**Published:** 2010-04-23

**Authors:** Hugh Mackenzie, Vivek Gulati, Samantha Tross

**Affiliations:** 1 Department of Orthopaedics, Ealing Hospital, Uxbridge Road, Southall, Middlesex UB1 3HW, UK

## Abstract

**Introduction:**

A synovial chondromatosis is a rare benign neoplasm on the synovium. Although described as a benign disease, it can be very destructive and can cause severe osteoarthritis and pain. To the best of our knowledge, we report the first known case of an extensive presentation of this intra-articular and extra-articular disease of the knee joint.

**Case presentation:**

A 49-year-old Caucasian man presented with right knee pain and stiffness caused by diffuse intra-articular and extra-articular synovial chondromatosis. He underwent careful preoperative imaging and planning followed by a two-stage arthroscopic and open procedure in order to completely eradicate the disease. He has regained full range of movement, but continues to experience residual pain due to severe osteoarthritis.

**Conclusions:**

Although synovial chondromatosis is described as a benign disease, it can be very destructive and debilitating. A challenging management dilemma arises when confronted with both synovial chondromatosis and osteoarthritis.

## Introduction

A synovial chondromatosis is a rare benign neoplasm that is caused by metaplasia of the synovium into chondrocytes [1]. The aetiology of the disease is uncertain. Milligram classified the disease into three phases: early (active intrasynovial disease but no loose bodies), transitional disease (active disease and loose bodies), and late (multiple loose bodies but no intrasynovial disease) [2].

The disease is commonly mono-articular and mostly affects the knee [3]. It occurs twice as frequently in men than women and usually presents with increasing joint pain and swelling during the third to fifth decade of a patient's life [4]. A patient with synovial chondromatosis experiences a decreased range of motion, palpable swelling, effusion, and crepitus [4].

The disease is usually intracapsular, but can also be extracapsular on rare occasions [5]. In this case report, we describe a patient with both intra- and extra-articular diseases. To the best of our knowledge, this is the first case with such an extensive presentation of intra- and extra-articular disease of the knee joint.

## Case presentation

A 49-year-old Caucasian man presented with a six-month history of progressively worsening right knee pain with associated swelling. The pain was present when the patient was at rest, and worsened when the leg was bearing weight, thus restricting his walking to short distances. His knee had become increasingly swollen. He denied any symptomatic night pain, locking, or a giving way of his knee. The patient was otherwise fit and well. His medical history was unremarkable and he was only taking ibuprofen for the pain.

Upon examination, the patient was seen to have marked quadriceps wasting of his right lower limb and a visibly swollen popliteal fossa. On palpation the swelling was hard, non-mobile, well defined, and measured 4 × 8 cm. The swelling was non-tender and there were no associated skin changes. Conversely, the patient had tenderness over the medial joint line. He could fully extend his knee, but flexion was restricted to only 115 degrees. There was no ligamentous instability and a McMurray test proved equivocal. An examination of the patient's hip revealed no abnormality.

**Figure 1:**
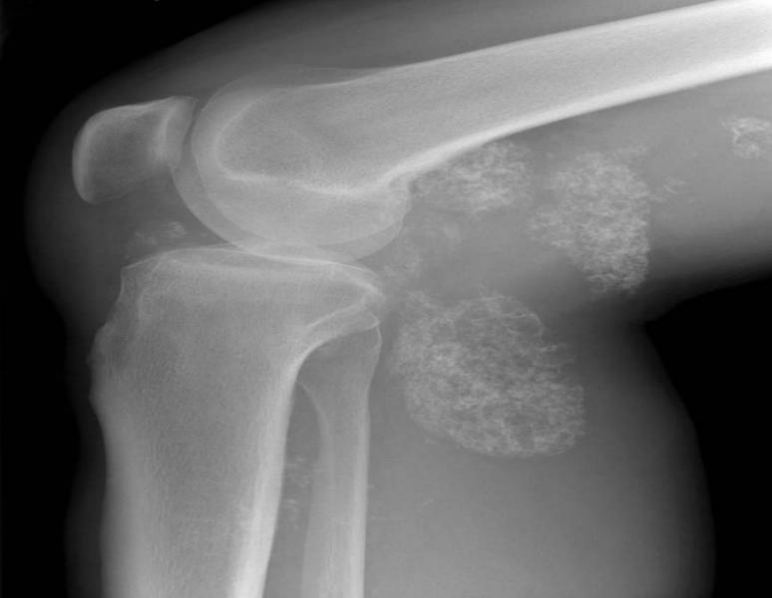
**Plain radiograph and magnetic resonance imaging scans showing multiple soft tissue calcifications within and outside the joint capsule of the right knee**.

A plain radiograph of the patient's knee revealed multiple calcific densities within the soft tissues surrounding it (Figure 1). Although some of these appeared to lie within the capsule, the majority appeared to be outside of it. These appearances were thought to be consistent with idiopathic tumoral calcinosis. However, to further scrutinize these calcifications, a magnetic resonance imaging (MRI) scan was recommended. It showed an extensive thickening of the patient's synovium, multiple intra-articular calcific and ossific loose bodies, and large calcified bursal extensions. The bursal component extended into the patient's posterior distal thigh and his proximal calf. These findings were thought to be consistent with very extensive synovial chondromatosis (Figure 1).

The patient's blood tests were normal: corrected calcium was 2.24 mmol/l, parathyroid hormone 2.5 pmol/l, inorganic phosphate 1.17 mmol/l, serum urate 296 μmol/l, white cell count 7.2 × 10^9^/l, and C-reactive protein 4 mg/l.

**Figure 2:**
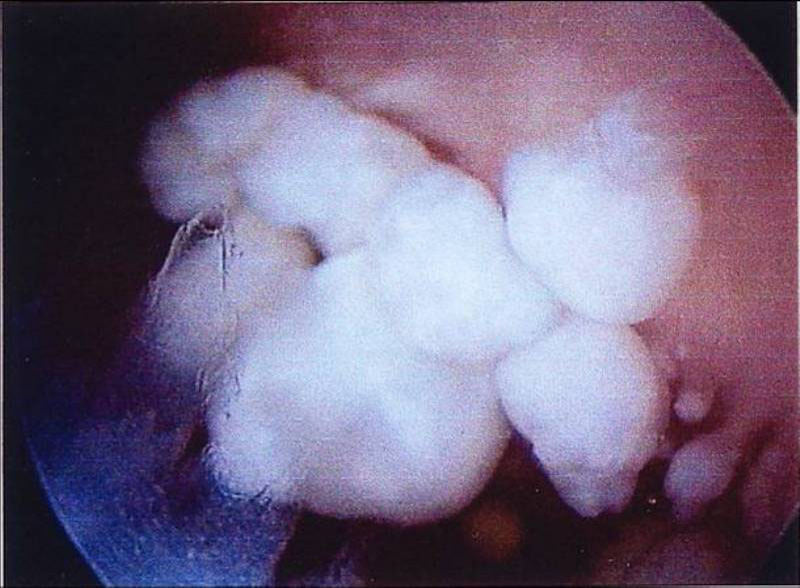
**An arthroscopic photograph showing nodules of chondromatosis fixed to the synovium**.

A two-stage procedure was planned following the findings of the MRI scan. The first stage was arthroscopy, which was able to note Grade IV osteoarthritis alongside florid synovial chondromatosis in the medial compartment (Figure 2). There were multiple loose bodies within this compartment and nodules were fixed to the synovium. A synovectomy with debridement and excision of these bodies was thus performed.

**Figure 3:**
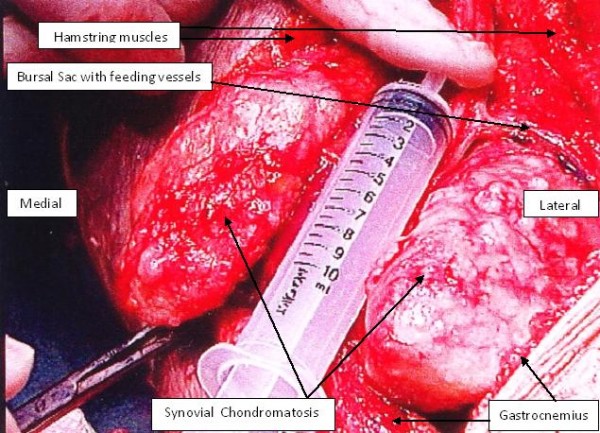
**An intraoperative photograph showing the extent of the popliteal disease**.

The second stage involved an open exploration of the patient's popliteal fossa. Three large calcified masses were found, all enclosed in bursal sacs (Figure 3). The first was just medial to the posterior tibial nerve; the second was deep into the medial head of the gastrocnemius muscle; and the third was lateral to the semimembranosus at the level of the oblique popliteal ligament. All three masses were excised and the sacs were closed with purse string sutures. A histological review at the Royal National Orthopaedic Hospital in Stanmore, UK confirmed our diagnosis of synovial chondromatosis. The sections showed nests of chondrocytes with focal ossification and focally attenuated synovium overlying the nodules.

After the operation, the patient underwent weekly physiotherapy sessions focusing on quadriceps strengthening, with a daily exercise regime to supplement this. He recovered well and three months after the operation, has regained his right knee's full range of movement with flexion increased to 130 degrees, which is equal to that of his left knee. He has residual medial joint line tenderness, undoubtedly due to osteoarthritis.

## Discussion

Cartilage cells are absent inside the synovial membrane. It follows therefore that the development of synovial chondromatosis depends on metaplastic transformation of the synovial cells into chondrocytes via an unknown stimulus [1]. These chondrocytes become pedunculated and encrusted inside the synovium and eventually expelled into the joint as loose bodies [6].

Extra-articular synovial chondromatosis is rare, but the combination of intra- and extra-articular diseases described here is an extremely rare condition. Given the initial X-ray image of large extra-articular calcification, we felt that the patient was more likely to have idiopathic tumoral calcinosis. However, tumoral calcinosis usually only affects people from Africa and the Caribbean in their second decade of life. Moreover, the calcifications are usually bilateral, affecting multiple sites, and are very rarely intra-articular [7]. Our patient, however, was Caucasian and the MRI scan showed a single lesion with an intra-articular component. Florid synovial chondromatosis was thus a more likely diagnosis. This was also confirmed by a histological examination.

Extra-articular diseases can be classified as tenosynovial chondromatosis or bursal chondromatosis depending on the origin [8]. In this case, we propose that either intra-articular synovial chondromatosis had penetrated the patient's popliteal bursas, or bursal chondromatosis had infiltrated his knee joint. To the best of our knowledge, this pattern of disease in the knee has only been reported twice in the literature and never to this extent [5,9]. This obviously raises concerns regarding a possible transformation to synovial chondrosarcoma. However, histological investigation revealed no significant nuclear atypia, thus ruling out malignancy. The literature reports only 33 cases of malignant transformation in the setting of histologically confirmed synovial chondromatosis [6]. A key feature of all these cases is the recurrence of benign disease prior to a diagnosis of malignant disease.

The extent of the disease and the presence of severe osteoarthritis also presented a challenging management problem. The combination of synovial chondromatosis and degenerative arthritis is a common finding in the advanced stage of the disease [3]. Primary synovial chondromatosis over time can lead to cartilage degeneration by mechanical wear via the loose bodies and through nutrient deprivation to the articular cartilage [3]. However, degenerative arthritis can lead to secondary synovial chondromatosis [3]. As radiotherapy and chemotherapy have no effect on synovial chondromatosis, surgical excision is the preferred treatment [4]. In cases that involve localized intra-articular disease, complete excision of the abnormal synovium seems to provide a cure. Generalized intra-articular disease with pain and swelling requires total synovectomy and a removal of the loose bodies. Extra-articular disease treatment aims for complete excision [10].

Three surgical options were considered, namely high tibial osteotomy (HTO), excision of the synovial and bursal chondromatosis alone, or excision combined with a total knee replacement. The ideal treatment for severe arthritis limited to the medial compartment in someone within the same age range as our patient is a unicompartmental knee replacement. However, without complete synovectomy, our patient's synovial chondromatosis could recur and thus compromise his joint replacement. HTO with realignment of the joint forces may lengthen the lifespan of the joint and delay the need for joint replacement. Total knee arthroplasty (TKA) has been proven to be an effective treatment for synovial chondromatosis. However, even with complete synovectomy alongside a TKA, recurrence of the disease has been reported [3]. This is probably due to incomplete synovectomy at the time of operation, which leaves remnants of pathological synovium [3]. Excision of the chondromatosis formed the initial surgical treatment plan, leaving us thus with the scope to perform an arthroplasty in should the need arise the future. To achieve full excision of the disease our patient required arthroscopic debridement to treat the intra-articular disease, as well as an open posterior approach to remove the disease from the popliteal bursas.

The residual pain experienced by the patient causes a further management dilemma. Although the pain is currently being controlled by analgesia, the possibility of HTO or TKA is being discussed with the patient.

## Conclusions

A synovial chondromatosis is a rare condition but one which can be highly aggressive and destructive. This case, with its rare presentation of intra- and extra-articular disease, highlights the importance of careful clinical assessment, lateral thinking, appropriate use of investigation, and careful pre-operative planning.

## Abbreviations

CRP: C-reactive protein; HTO: high tibial osteotomy; MRI: magnetic resonance imaging; TKA: total knee arthroplasty.

## Consent

Written informed consent was obtained from the patient for publication of this case report and any accompanying images. A copy of the written consent is available for review by the Editor-in-Chief of this journal.

## Competing interests

The authors declare that they have no competing interests.

## Authors' contributions

ST was the operating surgeon involved in the case. HM was the major contributor in writing the manuscript. VG edited the manuscript and assisted in reviewing the literature. All authors read and approved the final manuscript.
